# Design and Preparation of Chitosan-Crosslinked Bismuth Ferrite/Biochar Coupled Magnetic Material for Methylene Blue Removal

**DOI:** 10.3390/ijerph17010006

**Published:** 2019-12-18

**Authors:** Xiaoxi Cai, Jiang Li, Yunguo Liu, Xinjiang Hu, Xiaofei Tan, Shaobo Liu, Hui Wang, Yanling Gu, Lerong Luo

**Affiliations:** 1 College of Art and Design, Hunan First Normal University, Changsha 410205, China; 2 School of Architecture and Art, Central South University, Changsha 410083, China; 3 College of Environmental Science and Engineering, Hunan University, Changsha 410082, China; 4 College of Environmental Science and Engineering, Central South University of Forestry and Technology, Changsha 410004, China; 5 College of Materials Science and Engineering, Changsha University of Science and Technology, Changsha 410114, China

**Keywords:** biochar, magnetic, composite, adsorption, methylene blue

## Abstract

Biochar obtained by pyrolysis of the fiber plant kenaf was mixed with bismuth ferrite (BiFeO_3_) in a chitosan-containing acetic acid solution, magnetized, and modified to prepare a chitosan-crosslinked BiFeO_3_/biochar coupled magnetic material. The adsorption properties of the composite were investigated using methylene blue dissolved in water, and the effects of external conditions, such as pH, methylene blue concentration, reaction time, and temperature, on the adsorption performance were studied. The adsorption data were fitted and analyzed with kinetic and isotherm models, and the results showed that the BiFeO_3_/biochar coupled magnetic material effectively adsorbed methylene blue. The amounts adsorbed onto this magnetic material increased with increasing initial methylene blue concentration, reaction time, and temperature, and the adsorption performance improved under neutral and alkaline conditions. The pseudo-first-order kinetic and Langmuir isotherm models satisfactorily fitted the adsorption data, showing that the adsorption of methylene blue involved both chemical and physical adsorption. The maximum adsorption capacity of methylene blue onto the BiFeO_3_/biochar coupled magnetic material reached 18.942 mg·g^−1^ at 25 °C, confirming the excellent dye binding activity of this material.

## 1. Introduction

Dyes often contain many refractory and toxic organic pollutants [1,2], among which azo compounds and aromatic amines show carcinogenic and teratogenic effects [3]. If not properly disposed but instead discharged directly into water bodies, they can cause serious damage to the water environment [4]. Methylene blue, a representative wastewater contaminant used in printing and dyeing [5], is a heterocyclic, basic, cationic dye with a positive charge and is widely used in chemical indicators, dyes, biostains, and pharmaceuticals [6]. After methylene blue is discharged into water, the water transparency is affected, causing damage to the ecosystem [7]. Methylene blue is a relatively stable compound, and because it is not readily biodegradable, it poses a significant hazard to the ecological environment and drinking water systems [8]. There are also indications that it is toxic [9], as short-term contact can cause breathing difficulty, eye burns, and other adverse reactions such as increased heart rate, vomiting, shock, jaundice, etc. [10]. In severe cases, it can lead to quadriplegia and tissue necrosis [11]. Methylene blue can also produce toxic byproducts [8]. Therefore, before methylene blue-containing wastewater is discharged, it must be effectively treated to protect the ecological environment and human health [12]. Among the many wastewater treatment technologies, the adsorption method has the advantages of simple operation and short treatment times [13]. Attaining a highly efficient adsorbent is key to improving methylene blue wastewater treatments [14].

Biochar is a material with a high carbon content, high porosity, strong adsorption capacity, and versatility [15]. The surface of biochar is populated by many oxygen-containing functional groups (e.g., hydroxyl, carboxyl, aldehyde, keto, and ester groups) and polycyclic aromatic hydrocarbons [16,17]. The pore structure of the original biomass remains after the pyrolysis and carbonization processes; therefore, the pore structure of the resulting biochar requires no further development. Biochar has a high adsorption capacity for various organic pollutants in water [18,19]. However, because biochar powder particles are extremely fine, it is difficult to effectively separate them from aqueous solutions, which is disadvantageous for biochar recycling [20]. Therefore, the goal of this experimental program was to magnetically treat biochar using an external magnetic field to recover the magnetic biochar material after adsorption [15,21].

A multiferroic material refers to a material exhibiting more than one of the characteristics of (reverse) ferroelectricity, (reverse) ferromagnetism, and iron elasticity [22,23,24,25]. At a certain temperature, a multiferroic functional material undergoes spontaneous magnetization and polarization reactions [26]. Sometimes, spontaneous strain reactions will also occur [27]. BiFeO_3_ is known as an extremely rare multiferroic material that exhibits both ferroelectricity and antiferromagnetism at 25 °C [23,28]. Loading BiFeO_3_ onto biochar could effectively increase the biochar magnetic properties. Although simple magnetization solves the problem of separation from aqueous solutions, some pores of the biochar will be blocked, and some adsorption sites will be occupied due to the introduction of magnetic particles [29,30]. This results in a reduction in the original adsorption performance. Therefore, magnetic biochar should be further modified and improved.

Chitosan is a derivative of chitin [31]. Its molecular structure contains a large amount of free amino and hydroxyl groups, and the hydrogen bond between the main chains constitutes a secondary structure [32]. These chitosan characteristics create ion exchange, chelation, and adsorption properties, enabling the capturing of ions, organic substances, and biomolecules [33].

In this study, experiments were performed in which biochar and BiFeO_3_ were added at a weight ratio of 1:1 to a chitosan-acetic acid solution. The reaction conditions were adjusted with a sodium hydroxide solution and glutaraldehyde as a crosslinking agent to obtain crosslinked chitosan-loaded biochar and BiFeO_3_ composites. Biochar magnetization was carried out to prepare a chitosan-crosslinked BiFeO_3_/biochar coupled magnetic material. Chitosan effectively combines the properties of biochar and BiFeO_3_ to produce a magnetic and robust composite. The physical and chemical properties and structural characteristics were analyzed, and the adsorption properties of the coupled material were studied in aqueous methylene blue solutions. The effects of pH, initial methylene blue concentration, reaction time, and temperature on the adsorption performance were investigated, and kinetic and isothermal models were used to elucidate the adsorption mechanism.

## 2. Materials and Methods

### 2.1. Material Preparation

#### 2.1.1. Preparation of Biochar

The raw material kenaf was rinsed with distilled water and dehydrated in a dry, ventilated place. The dried kenaf was further baked in an oven at 70 °C until completely free of water, placed in a pulverizer, and ground into a powder, after which the powder was sieved through a 100 mesh grid. The sieved powder was placed in a quartz boat, and the quartz boat was transferred to a quartz tube in a tubular electric furnace. After sealing, the heating rate of the electric furnace was set to 5 °C·min^−1^. After reaching the target temperature of 500 °C, the temperature was kept constant until pyrolysis reached completion (2 h). The resulting material was cooled to 25 °C. After grinding, the sieved biochar was stored in a sealed bag for further use.

#### 2.1.2. Preparation of BiFeO_3_

Ferric nitrate, bismuth nitrate, and citric acid (0.08 mol of each) were weighed; the solid ferric nitrate and bismuth nitrate were dissolved in 200 mL of ethylene glycol methyl ether, and 0.2 mL of a 0.1 mol·L^−1^ nitric acid solution was added. Then, the weighed citric acid was dissolved in 100 mL of ethylene glycol, and the two solutions were evenly mixed. The vessel containing the mixture was heated in a water bath at 60 °C for 1 h to obtain a light-brown gel, which was then placed in a crucible. The crucible was placed in a muffle furnace (Junke, Shanghai, China) and preheated to 200 °C. After 0.5 h, the reaction temperature was set to 500 °C. After calcination for 2 h, the sample was cooled and ground to obtain the finished BiFeO_3_ material.

#### 2.1.3. Synthesis of BiFeO_3_/Biochar Coupled Magnetic Material

**Figure 1:**
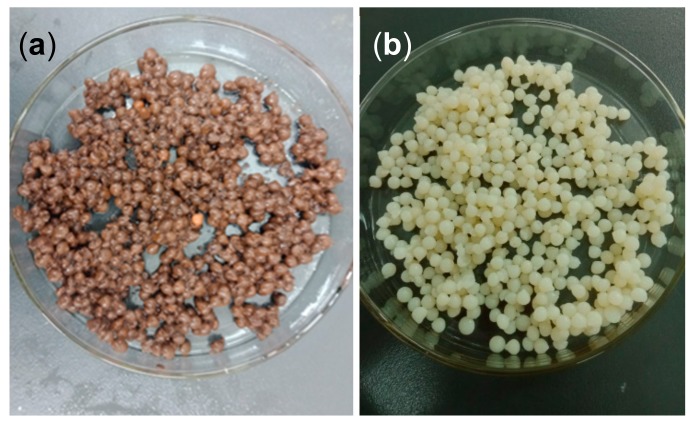
BiFeO_3_/biochar coupled magnetic beads (**a**) and blank beads (**b**).

A volume of 1 mL of acetic acid was placed into a 100 mL volumetric flask and diluted with ultrapure water to obtain a 1% (by volume) acetic acid solution. Then, 2 g of chitosan was dissolved in the acetic acid solution to obtain a 2% (by weight) chitosan-acetic acid solution. Biochar (2 g) and BiFeO_3_ (2 g) were added to the solution at 25 °C and magnetically stirred until homogeneous. The resulting mixed solution was slowly dropped with a syringe into a 1% NaOH solution to form chitosan gel beads with a uniform particle size. After standing for 4 h, the chitosan gel beads were removed from the solution and washed, then placed in 400 mL of deionized water, and 5 mL of a glutaraldehyde aqueous solution was added. After adjusting the pH to 9.0, the mixture was slowly stirred at 60 °C for 2 h to induce cross-linking and finally allowed to stand for 20 h. The beads were removed and washed to neutral to obtain the final crosslinked chitosan-loaded BiFeO_3_/biochar composite. The above steps were repeated without adding biochar or BiFeO_3_ to make blank beads. The obtained finished products are shown in Figure 1.

### 2.2. Characterization Methods

X-ray diffraction (XRD) patterns of the materials were measured using a D/max-2500 (Rigaku, Tokyo, Japan) with Cu Kα radiation. The microstructures of the materials were characterized by scanning electron microscopy (SEM; QUANTA 250 FE-SEM, FEI, Hillsboro, OR, USA) in ETD morphology mode under a 20 kV working voltage and 0° angle. The surface elements were analyzed using an ESCALAB 250Xi X-ray photoelectron spectrometer (XPS, Thermo Fisher Scientific, Waltham, MA, USA), and the magnetic properties of the samples were measured with a MPMS-XL-7 vibrating sample magnetometer (Quantum Design Instruments, O’Fallon, MO, USA). Fourier transform infrared (FTIR) analyses were performed on a NICOLET 5700 Fourier transform IR spectrometer (Thermo Nicolet Corporation, Madison, WI, USA) across an acquisition range of 400–4000 cm^−1^. Thermogravimetric (TG) curves were measured using an SDT Q600 thermal analyzer (TA, Westlake, OH, USA) with heating from 25 to 1000 °C (flux rate 100 mL·min^−1^, heating rate 5 °C·min^−1^). The Brunner–Emmet–Teller (BET) specific surface area of the material was measured using a Micromeritics 3Flex analyzer (Micromeritics Instrument Corporation, Norcross, GA, USA).

### 2.3. Adsorption Experiments

#### 2.3.1. Effect of pH on Adsorption

Methylene blue solutions were prepared (concentration of 50 mg·L^−1^) with pH values from 3.0 to 9.0, and 50 mL aliquots were transferred into a 150 mL conical flask. BiFeO_3_/biochar coupled magnetic beads (wet weight: 2 g, dry weight: 0.1299 g) were subsequently added, and the flask was sealed with plastic wrap. The reaction temperature was set to 25 °C, and the sample was placed for 4 h in a constant-temperature oscillator with a rotation speed of 170 rpm. The sample was analyzed thereafter.

#### 2.3.2. Effect of Adsorbent Dose on Adsorption

Methylene blue solutions were prepared (concentration of 50 mg·L^−1^) at pH 6.0, and 50 mL aliquots were transferred into a 150 mL conical flask. BiFeO_3_/biochar coupled magnetic beads (wet weight 1 to 8 g) were subsequently added, and the flask was sealed with plastic wrap. The reaction temperature was set to 25 °C, and the sample was placed for 4 h in a constant-temperature oscillator with a rotation speed of 170 rpm. The sample was analyzed thereafter.

#### 2.3.3. Effect of Initial Methylene Blue Concentration C_0_ and Reaction Temperature on Adsorption

Methylene blue solutions with initial concentrations of 10, 20, 40, 60, 80, and 100 mg·L^−1^ were prepared, and 50 mL of each was transferred into a 150 mL conical flask. The pH was adjusted to 6.0, 2 g of BiFeO_3_/biochar coupled magnetic beads was added, and the flask was sealed with plastic wrap. The reaction temperature was set to 15, 25, or 35 °C, the wrapped flask was placed for 4 h in a constant-temperature oscillator with a rotation speed of 170 rpm. The mixture was thereafter sampled and analyzed.

#### 2.3.4. Effect of Reaction Time on Adsorption

A methylene blue solution with an initial concentration of 50 mg·L^−1^ was prepared, and a 150 mL aliquot was placed into a 250 mL conical flask. The pH of the solution was adjusted to 6.0, 6 g of BiFeO_3_/biochar coupled magnetic beads was added to the solution, and the flask was sealed with plastic wrap. The reaction temperature was set to 25 °C, and the flask was placed in a constant-temperature oscillator with the speed adjusted to 170 rpm. The time intervals for the kinetic test were 10 min, 20 min, 25 min, 40 min, 50 min, 60 min, 90 min, 120 min, 180 min, and 240 min.

#### 2.3.5. Regeneration and Reuse Experiments

In order to test the regeneration and reuse capability of the BiFeO_3_/biochar coupled magnetic material, a 1.0 mol·L^−1^ CH_3_COOH solution was used to desorb the adsorbed methylene blue. Then, the regenerated BiFeO_3_/biochar coupled magnetic material was washed to neutral and reused for further adsorption experiments.

## 3. Results

### 3.1. Characterization

**Figure 2:**
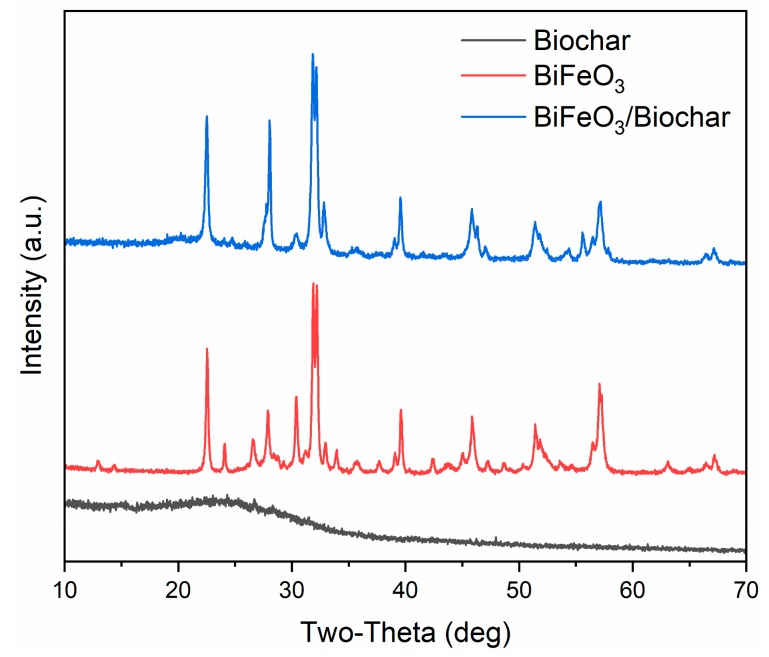
X-ray diffraction patterns of biochar, BiFeO_3_, and BiFeO_3_/biochar coupled magnetic material.

Figure 2 shows the XRD patterns of the biochar, BiFeO_3_, and BiFeO_3_/biochar coupled magnetic material. For BiFeO_3_, the major peaks assigned to the (012), (104), (110), (006), (202), (024), (116), (122), (018), (202), (214), (300), (208), and (220) planes indicated planar BiFeO_3_ with a single-phase perovskite structure (JCPDS No. 20-169) [27,34]. The biochar exhibited a broad diffraction peak near 24°, which suggested that it contained amorphous carbon [35]. In the XRD pattern of the BiFeO_3_/biochar coupled magnetic material, the main peaks for biochar and BiFeO_3_ were observed, and it was confirmed that the synthesized BiFeO_3_/biochar coupled magnetic material contained different amounts of biochar and BiFeO_3_.

**Figure 3:**
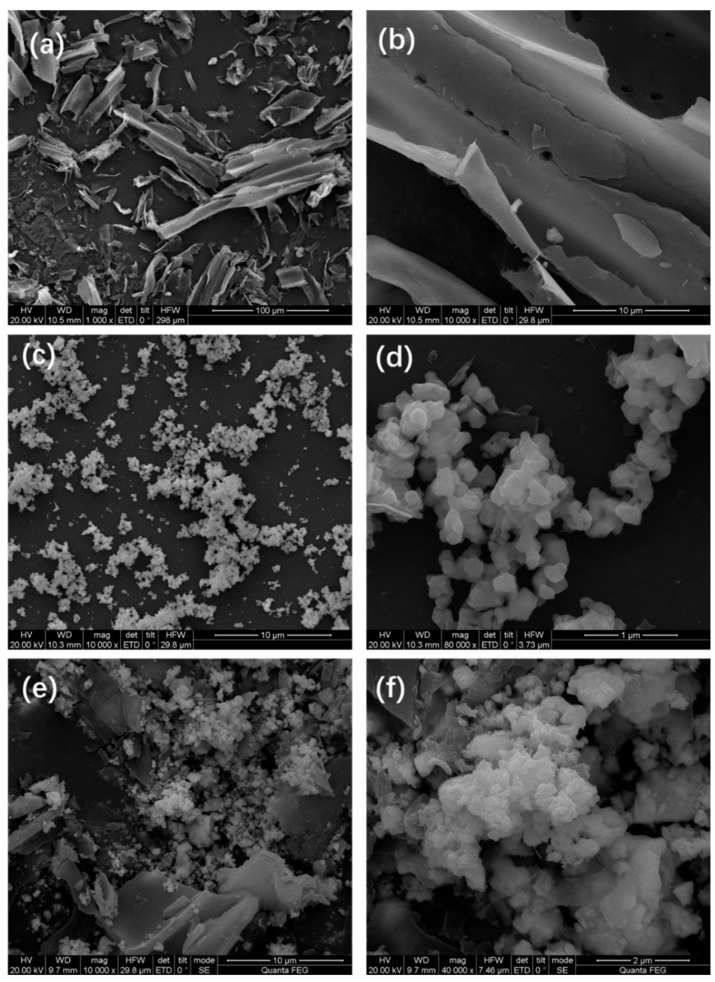
Scanning electron microscopy images of (**a**,**b**) biochar, (**c**,**d**) BiFeO_3_, and (**e**,**f**) BiFeO_3_/biochar coupled magnetic material.

**Figure 4:**
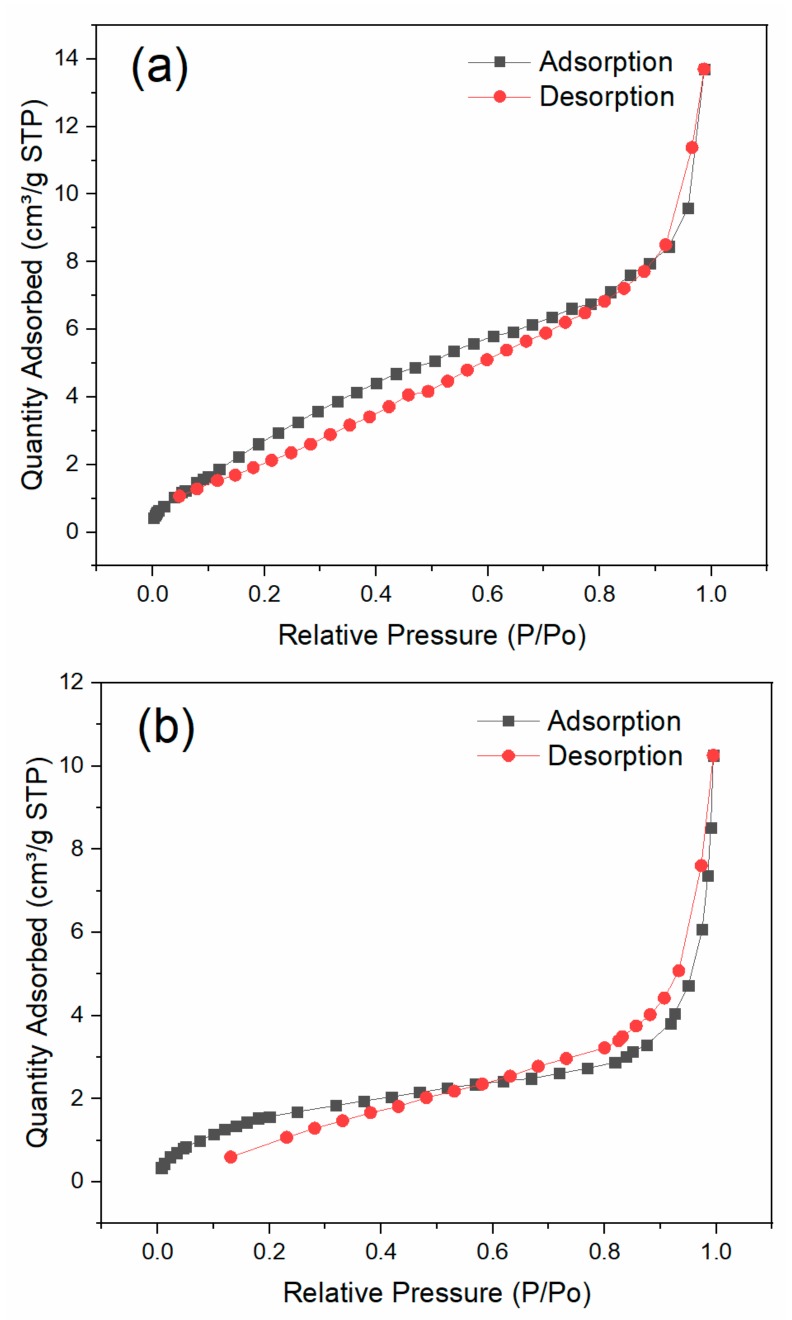
Nitrogen adsorption–desorption isotherms of (**a**) BiFeO_3_ and (**b**) BiFeO_3_/biochar.

The SEM morphologies of the prepared biochar, BiFeO_3_, and BiFeO_3_/biochar coupled magnetic material are shown in Figure 3. As shown in Figure 3a,b, the surface of the biochar contained numerous pores. In contrast, BiFeO_3_ consisted of fine particles with a grain size of approximately 100 nm. For the BiFeO_3_/biochar coupled magnetic material (Figure 3e,f), the introduction of biochar resulted in a change in the morphology of BiFeO_3_, where the BiFeO_3_ nanoparticles were randomly distributed on the surface of the biochar sheet. The N_2_ adsorption–desorption isotherms of BiFeO_3_ and BiFeO_3_/biochar are shown in Figure 4, which resembled the shape of Type II isotherms [36]. The BET specific surface area of BiFeO_3_ was 14.09 m^2^·g^−1^, and that of the BiFeO_3_/biochar coupled magnetic material was 6.78 m^2^·g^−1^. This decrease in surface area is mainly due to the small specific surface area of the biochar and clogging of the biochar pores by BiFeO_3_. 

**Figure 5:**
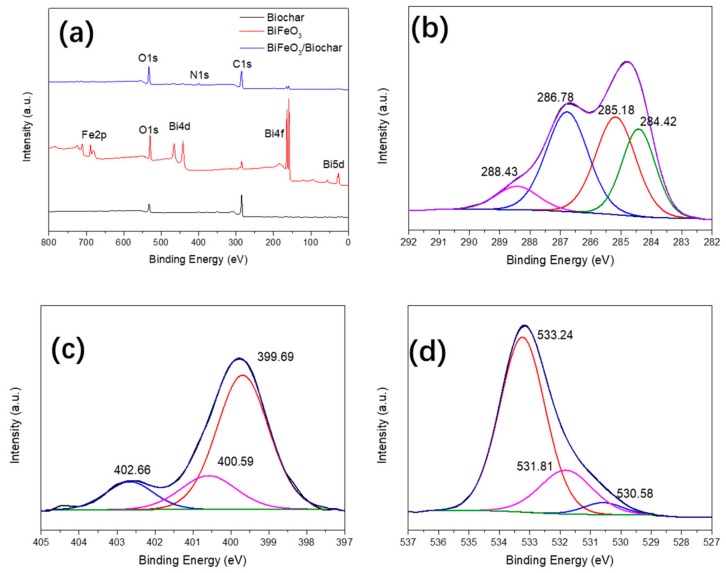
(**a**) X-ray photoelectron spectrometer plots of biochar, BiFeO_3_, and BiFeO_3_/biochar coupled magnetic material; high-resolution (**b**) C1s, (**c**) N1s, and (**d**) O1s spectra of BiFeO_3_/biochar coupled magnetic material.

To study the combined state and elemental compositions of the biochar, BiFeO_3_, and BiFeO_3_/biochar coupled magnetic material, we performed XPS analysis (Figure 5). As shown in Figure 5a, XPS analysis of the BiFeO_3_/biochar coupled magnetic material showed that the main elements of the prepared sample were Bi, O, Fe, C, and N. The C element in the composite material can be attributed to biochar and chitosan; the O element corresponds to chitosan, BiFeO_3_, and biochar; and Bi and Fe originate from BiFeO_3_. Figure 5b shows the C1s XPS results of the BiFeO_3_/biochar coupled magnetic material obtained at a high resolution. The spectrum can be fitted to four different peaks at 284.42, 285.18, 286.78, and 288.43 eV corresponding to C–C (biochar and chitosan), N–C (chitosan), C–O (biochar and chitosan), and C=O (biochar and chitosan), respectively [37,38]. The N1s spectrum in Figure 5c shows three peaks at 399.69, 400.59, and 402.66 eV corresponding to –NH_2_, –NHCO–, and C–N, respectively [39,40]. The O1s spectrum in Figure 5d has three characteristic peaks at 530.58, 531.81, and 533.24 eV corresponding to Fe–O, C–O, and C=O, respectively [41]. These results indicate that biochar and BiFeO_3_ were successfully supported on the chitosan carrier.

**Figure 6:**
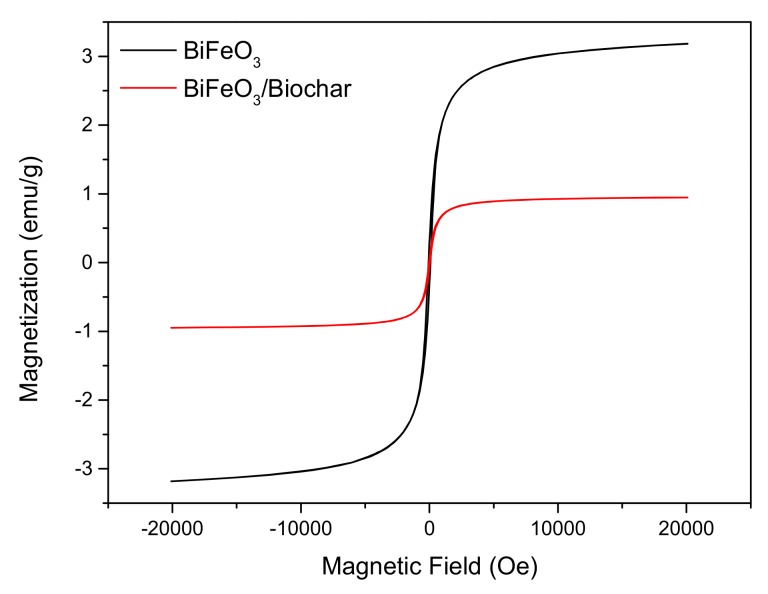
Magnetization curves of BiFeO_3_ and BiFeO_3_/biochar coupled magnetic material.

In order to study the magnetic properties, the magnetization hysteresis curves of the BiFeO_3_ and BiFeO_3_/biochar-coupled magnetic material were measured by vibrating sample magnetometry (Figure 6). The hysteresis loops are sigmoid curves. The saturation magnetization (*M*_s_), coercive force (*H*_c_), and remanence (*M*_r_) of BiFeO_3_ were 3.18 emu·g^−1^, 78.36 Oe, and 0.31 emu·g^−1^, respectively, and those of the BiFeO_3_/biochar coupled magnetic material were 0.95 emu·g^−1^, 43.21 Oe, and 0.08 emu·g^−1^, respectively. The remanence of both samples was very small (close to zero), indicating that the magnetization almost disappeared when the external magnetic field was removed. The BiFeO_3_ and BiFeO_3_/biochar coupled magnetic material exhibit superparamagnetism at 25 °C, which is important for convenient recycling of these composites [42,43]. After completion of the adsorption process, the BiFeO_3_/biochar coupled magnetic material can be collected and separated from the aqueous solution using a magnet. 

**Figure 7:**
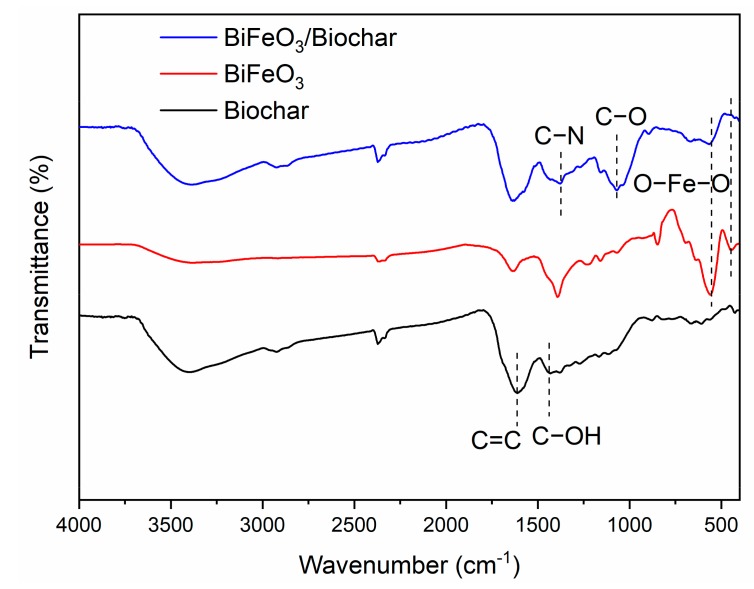
Fourier transform infrared spectra of biochar, BiFeO_3_, and BiFeO_3_/biochar magnetic material.

Figure 7 shows the FTIR spectra of the biochar, BiFeO_3_, and BiFeO_3_/biochar magnetic material. For the biochar, the absorption peak at 1426 cm^−1^ corresponds to the stretching vibration of the C−OH group, and the peak at 1612 cm^−1^ corresponds to the C=C stretching vibration mode. For BiFeO_3_, two strong peaks were observed at 438 and 552 cm^−1^ corresponding to the O−Fe−O bending vibration in BiFeO_3_ and Fe−O stretching of the FeO_6_ group, respectively. In the FTIR spectrum of the BiFeO_3_/biochar coupled magnetic material, the absorption peak at approximately 1378 cm^−1^ originates mainly from C−N in chitosan [41], and the absorption peak at 1067 cm^−1^ corresponds to C−O in the chitosan coupling material. In addition, all peaks corresponding to biochar and BiFeO_3_ were also present in the coupling material spectrum, which further indicates that chitosan effectively binds biochar and BiFeO_3_.

**Figure 8:**
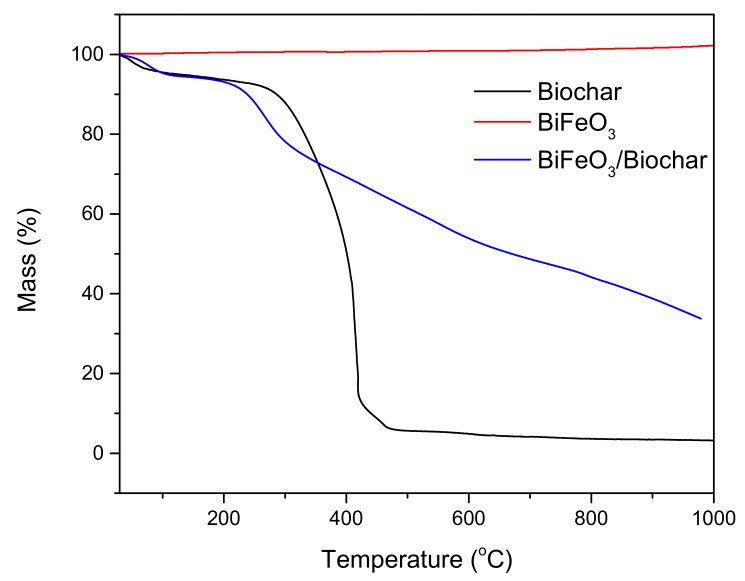
Thermogravimetric curves of biochar, BiFeO_3_, and BiFeO_3_/biochar coupled magnetic material.

The results of the TG analysis of biochar, BiFeO_3_, and BiFeO_3_/biochar coupled magnetic material are shown in Figure 8. Under an air atmosphere and heating to 1000 °C, the biochar lost approximately 96.79% of its total mass. The mass reduction by 4.89% in the interval of 25–120 °C is mainly due to the dehydration process [44]. When the temperature was gradually increased from 120 °C to 500 °C, the mass of the biochar was significantly reduced by 88.48% owing to destruction of the biochar structure. No significant mass loss was observed for BiFeO_3_. The mass loss of the BiFeO_3_/biochar coupled magnetic material at 1000 °C represents 66.25% of its total mass.

### 3.2. Effect of pH

**Figure 9:**
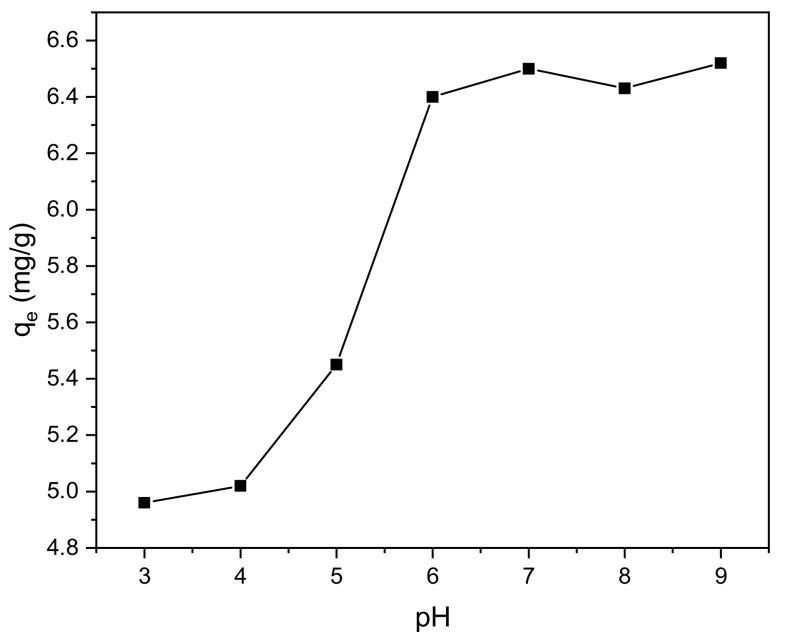
Effect of pH on the adsorption process (*V* = 0.05 L, *T* = 25 °C, *t* = 240 min, *m* = 0.1299 g).

The effect of pH change on adsorption is shown in Figure 9, illustrating that the adsorption and removal rate of methylene blue by the BiFeO_3_/biochar coupled magnetic beads were affected by pH. The adsorption increased with increasing pH. When the pH of the reaction solution was raised from 3.0 to 6.0, the adsorption amount and removal rate were markedly improved. When the pH was further raised from 6.0 to 9.0, the changes observed in the adsorption and removal rate were not as significant.

This observation is mainly related to the surface charge of the adsorbent material as the pH of the solution changes. When the pH of the reaction solution is 3.0, the solution is strongly acidic with a high concentration of H^+^ cations present. Methylene blue is a cationic dye and thus also has a positive charge. The presence of H^+^ cations together with positively charged methylene blue in the solution results in competitive adsorption. When the pH is high, the solution is alkaline, and the carboxyl groups on the surface increase the negative charge of the biochar due to deprotonation [45]. Therefore, more charged adsorption sites are generated to adsorb methylene blue, resulting in a higher degree of methylene blue adsorption onto the modified biochar beads. The above trend indicates that the BiFeO_3_/biochar coupled magnetic material is effective in removing methylene blue under neutral and alkaline conditions.

### 3.3. Effect of Adsorbent Dose

**Figure 10:**
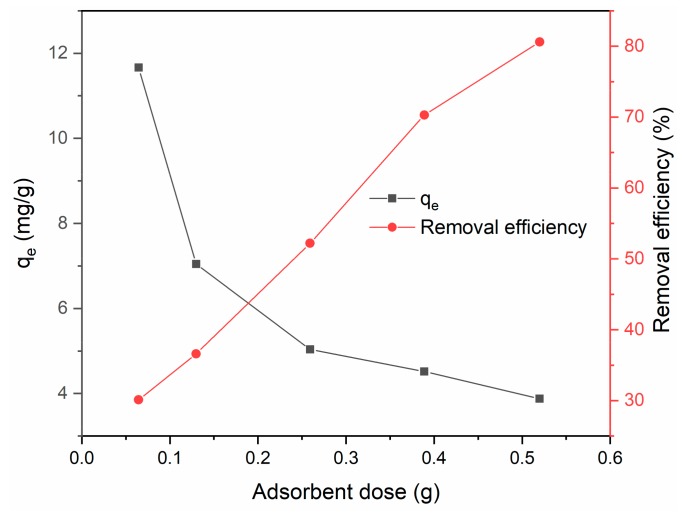
Effect of adsorbent dose on adsorption efficiency and capacity (*V* = 0.05 L, *T* = 25 °C, *t* = 240 min, pH = 6).

The effects of adsorbent dose on adsorption efficiency and capacity are shown in Figure 10. The removal efficiency increased with increasing adsorbent dose, which was attributed to an increased amount of available adsorption sites. However, the adsorption capacity *q*_e_ of the BiFeO_3_/biochar coupled magnetic material decreased with increasing adsorbent dose. This might be because excess adsorbent provides excess adsorption sites and thus reduces the adsorbent utilization efficiency.

### 3.4. Effect of Initial Methylene Blue Concentration C_0_ and Reaction Temperature

**Figure 11:**
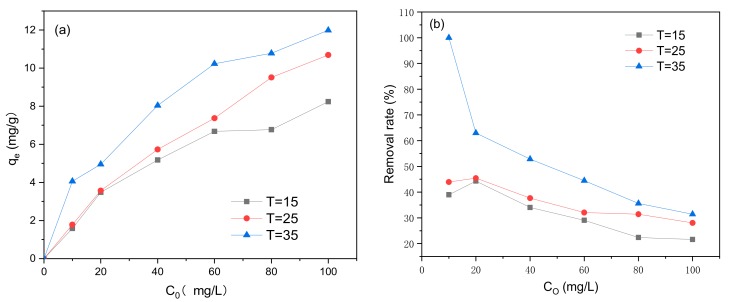
Effect of initial methylene blue concentration *C*_0_ and reaction temperature on (**a**) adsorption capacity and (**b**) removal efficiency (*V* = 0.05 L, pH = 6.0, *t* = 240 min, *m* = 0.1299 g).

The effects of initial methylene blue concentration *C*_0_ and reaction temperature on adsorption are shown in Figure 11. The adsorption of methylene blue using the modified biochar was affected by the temperature at which the reaction was carried out, where the adsorption amount and the removal rate were higher at higher temperatures. Based on Figure 11, as *C*_0_ increased, the concentration of methylene blue adsorbed also increased; however, the removal rate decreased.

According to the solvent displacement theory for liquid-phase adsorption, the diffusion rate of methylene blue is affected by temperature changes and the diffusion speed [46]. A temperature increase or viscosity decrease results in an increase in the adsorption rate and adsorption equilibrium concentration. This indicates that the adsorption of methylene blue onto the BiFeO_3_/biochar coupled magnetic material is an endothermic reaction. When the concentration of methylene blue equals the upper limit of the number of adsorption sites, the adsorption reaches equilibrium because the number of adsorption sites is constant. Therefore, if the concentration of methylene blue subjected to the adsorption reaction increases, the corresponding removal rate decreases.

### 3.5. Effect of Reaction Time

**Figure 12:**
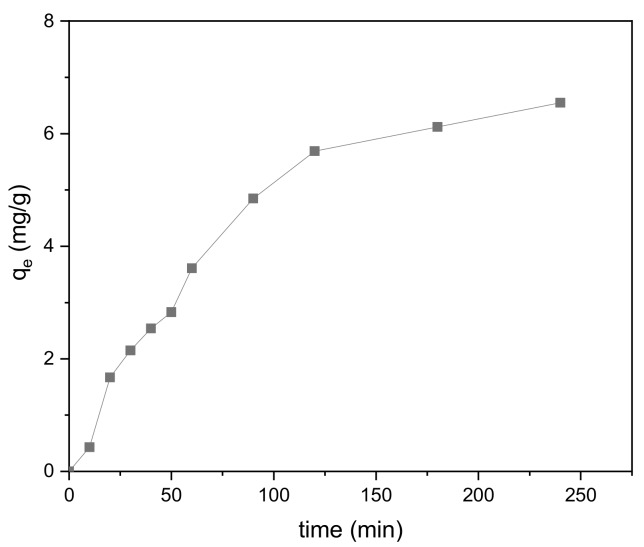
Effect of reaction time on the adsorption process (*V* = 0.15 L, pH = 6.0, *T* = 25 °C, *m* = 0.3889 g).

The effect of reaction time on adsorption is shown in Figure 12. From zero to 120 min, the adsorption amount increased significantly. Between 120 and 180 min, the rate of increase in adsorption became noticeably lower, and it began to flatten out during the last 180 to 240 min. The entire process is consistent with the characteristics of liquid-phase adsorption on porous adsorbents. In addition, the adsorption of methylene blue onto the modified biochar reached equilibrium at a relatively high rate, indicating that the adsorption is mainly physical in nature.

In the early stage of adsorption, there are many vacant sites on the surface of the BiFeO_3_/biochar coupled magnetic beads, which explains the faster adsorption of methylene blue. At later reaction times, as the adsorption sites become gradually occupied, repulsion between the methylene blue molecules occurs, resulting in a stable state of adsorption and desorption and indicating that the adsorption reached equilibrium.

### 3.6. Adsorption Isotherms

**Figure 13:**
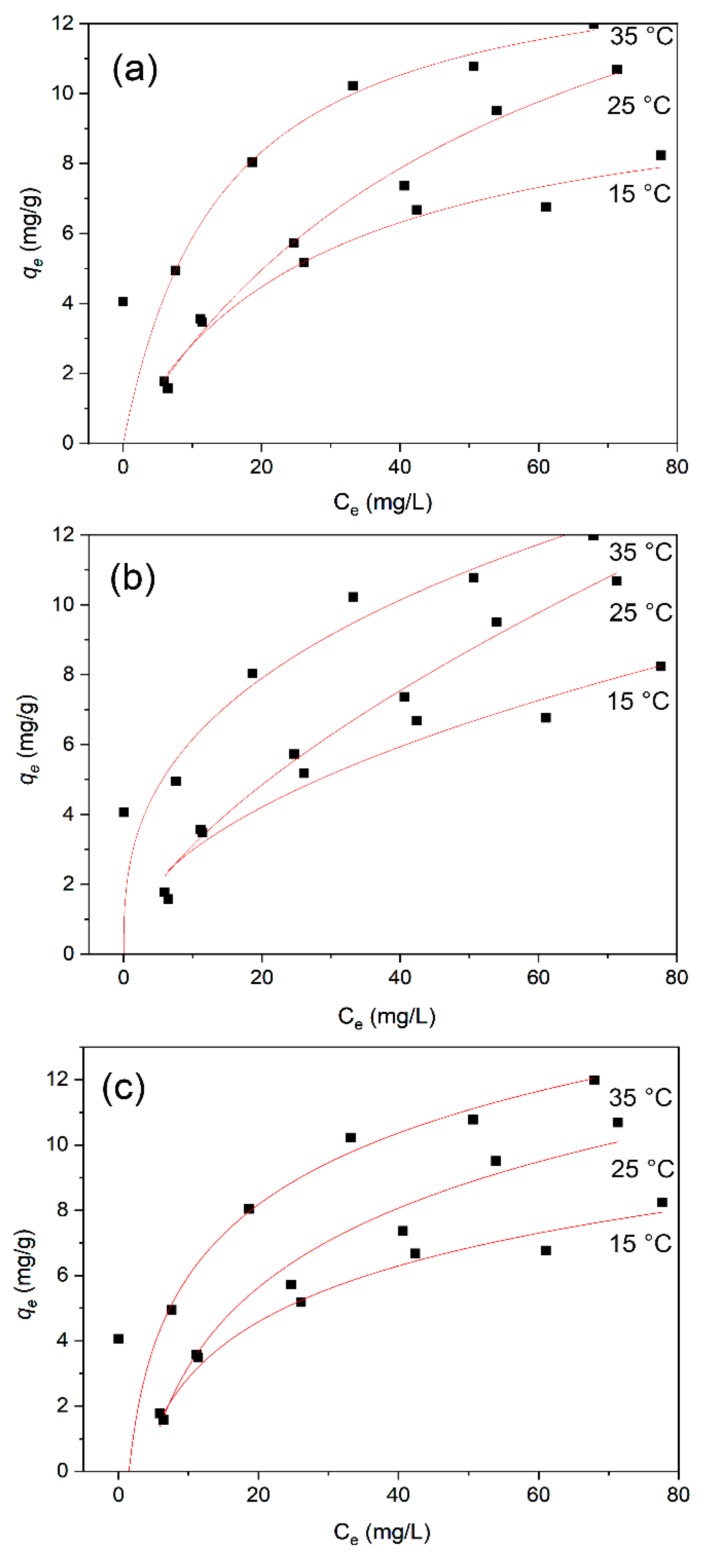
Isotherm model data for methylene blue adsorption onto BiFeO_3_/biochar coupled magnetic material: (**a**) Langmuir, (**b**) Freundlich, and (**c**) Temkin models.

In order to investigate the mechanism and fundamentals of methylene blue adsorption onto the BiFeO_3_/biochar coupled magnetic beads, the experimental data were fitted with three isothermal models: Langmuir, Freundlich, and Temkin [47]. The data of the relevant adsorption isotherms obtained after fitting were analyzed, and the results are shown in Figure 13. The expressions for the three adsorption isotherms are as follows:

*q_e_* = *q_max_K_L_C_e_*/(1 + *K_L_C_e_*), (1)

*q_e_* = *K_F_C_e_*^(1/*n*)^, (2)

*q*_e_ = *RT* *ln(*a*_T_*C*_e_)/*b*_T_, (3)

where *q_e_* is the amount of adsorbed methylene blue (mg·g^−1^); *q_max_* is the maximum adsorption amount (mg·g^−1^); *K_L_* is the Langmuir adsorption constant (L·mg^−1^), which is related to the affinity of the adsorption sites; *C_e_* is the concentration measured at the end of the adsorption reaction (mg·L^−1^); *K_F_* is the unit capacity constant of the Freundlich model (L·mg^−1^); *n* is the Freundlich constant related to the adsorption strength; *R* is the ideal gas constant with a value of 8.314 × 10^−3^ kJ·mol^−1^·K^−1^; *T* is the absolute temperature (K); and *a_T_* (L·g^−1^) and *b_T_* (kJ·mol^−1^) are the Temkin constants. The relevant parameters obtained from the three adsorption isotherms fitted with the Langmuir, Freundlich, and Temkin models are given in Table 1. 

Comparing *R*^2^ values, the Langmuir adsorption isotherm model is more consistent with the data of this adsorption experiment, which indicates that the BiFeO_3_/biochar coupled magnetic material has a uniform surface and uniformly distributed adsorption sites. The good fit of the model indicates that a single-layer adsorption reaction may occur because the Langmuir adsorption isotherm model is based on the assumption that only monolayer adsorption occurs on the surface of the adsorbent [48,49]. In addition, the parameters of the Temkin isotherm adsorption model show that the binding energies (*b_T_*) in the three systems in this study are 0.969, 0.707, and 0.811 kJ·mol^−1^. These values are not representative of ion exchange adsorption nor physical adsorption, meaning that the adsorption of methylene blue onto the BiFeO_3_/biochar coupled magnetic material involves both chemical adsorption and physical adsorption.

The maximum amount of adsorbed methylene blue by the adsorbent in this study, which reached 18.942 at 25 °C, is not the highest in all published literatures (Table 2). However, the BiFeO_3_/biochar coupled magnetic material prepared in this research has a good adsorption efficiency and faster separation effect from aqueous solutions than other materials, as well as a better recycling efficiency caused by the same. At the same time, it can effectively avoid secondary pollution to the environment.

The thermodynamic parameters calculated at three different temperatures (15, 25, and 35 °C) are shown in Table 3. The changes in free energy (∆*G*^0^) at 15, 25, and 35 °C were −0.783, −0.820, and −1.748 kJ·mol^−1^, respectively. The free energy changes were all negative, indicating that the adsorption process of methylene blue by the BiFeO_3_/biochar coupled magnetic material is spontaneous. The ∆*G*^0^ values decreased with increasing temperature, indicating that a high temperature was favorable for adsorption. The enthalpy change (Δ*H*^0^ = 26.857 kJ·mol^−1^) obtained in this study was positive, indicating that the adsorption process of methylene blue by the BiFeO_3_/biochar coupled magnetic material is endothermic.

### 3.7. Adsorption Kinetics

**Figure 14:**
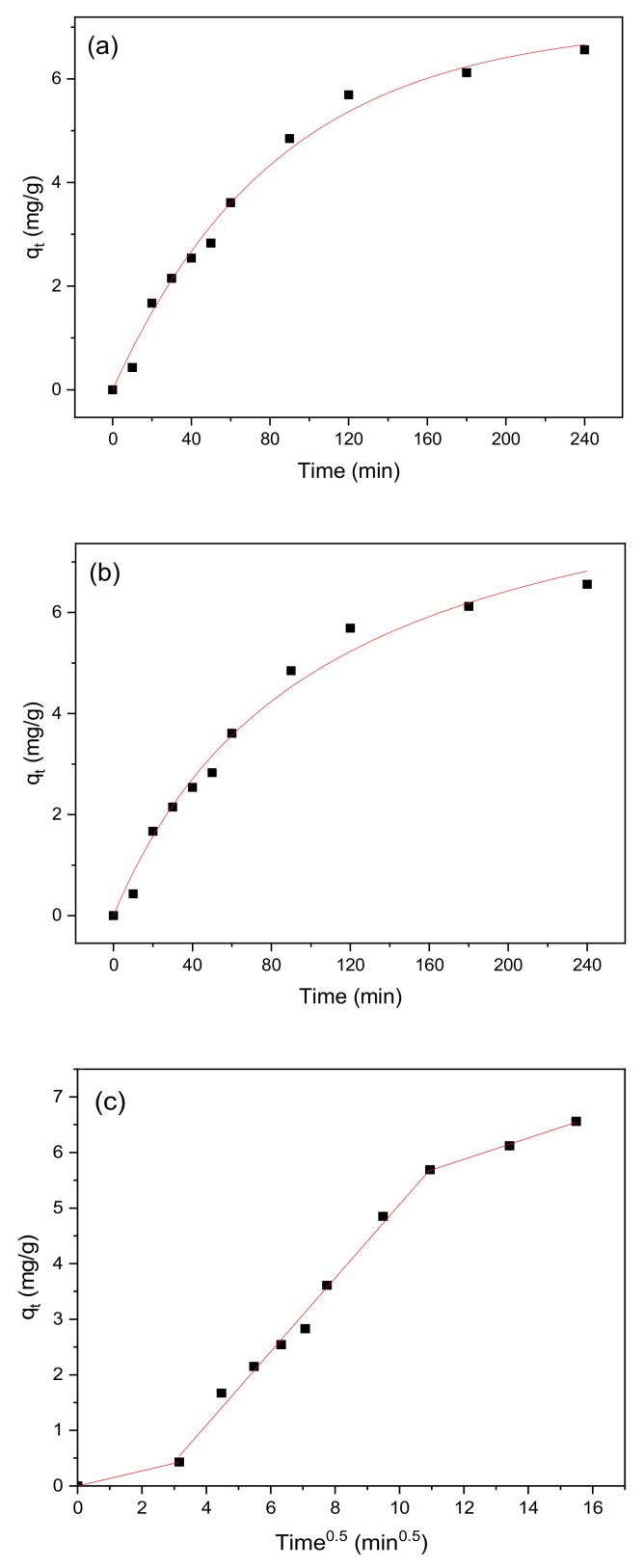
(**a**) Pseudo-first-order, (**b**) pseudo-second-order, and (**c**) intra-particle diffusion model curves for methylene blue adsorption onto BiFeO_3_/biochar coupled magnetic material.

The pseudo-first-order model, pseudo-second-order model, and intra-particle diffusion model were used to fit the experimental data of the adsorption reaction [56] to further investigate the adsorption mechanism of methylene blue onto the BiFeO_3_/biochar coupled magnetic material. The results are shown in Figure 14, and the expressions of the three adsorption kinetic models are as follows:

*q_t_* = *q_e_*(1 − *e*^−*k*1*t*^), (4)

*q_t_* = *q*_*e*_^2^*k*_2_*t*/(1 + *q_e_k*_2_*t*), (5)

*q_t_* = *k_p_t*^0.5^+*C*, (6)

where *q_e_* and *q_t_* are the adsorption amounts of methylene blue at equilibrium and at time *t* (min), respectively (mg·g^−1^); *k*_1_ is the pseudo-first-order rate constant (min^−1^); *k*_2_ is the pseudo-second-order rate constant (g·mg^−1^·min^−1^); and *k_p_* is the diffusion rate constant (mg·g^−1^·min^−0.5^).

The relevant parameters obtained from the pseudo-first-order and pseudo-second-order models are shown in Table 4. By comparing *R*^2^, the pseudo-first-order model provides a better fit of the results from methylene blue adsorption onto the BiFeO_3_/biochar coupled magnetic material. It could also be confirmed that this adsorption reaction includes physical adsorption [56]. The most critical factor determining the overall rate of adsorption is attributed to the slowest step in the process. As shown in Figure 14, the graph obtained with the intra-particle diffusion model consists of three linear segments, indicating that the adsorption process has three rate-controlling steps. The first step is representative of the membrane diffusion process, where methylene blue is transferred from the solution to the surface of the BiFeO_3_/biochar coupled magnetic material. The second step corresponds to internal particle diffusion, and the third step is indicative of adsorption equilibrium [57].

### 3.8. Reuse of BiFeO_3_/Biochar Coupled Magnetic Material

**Figure 15:**
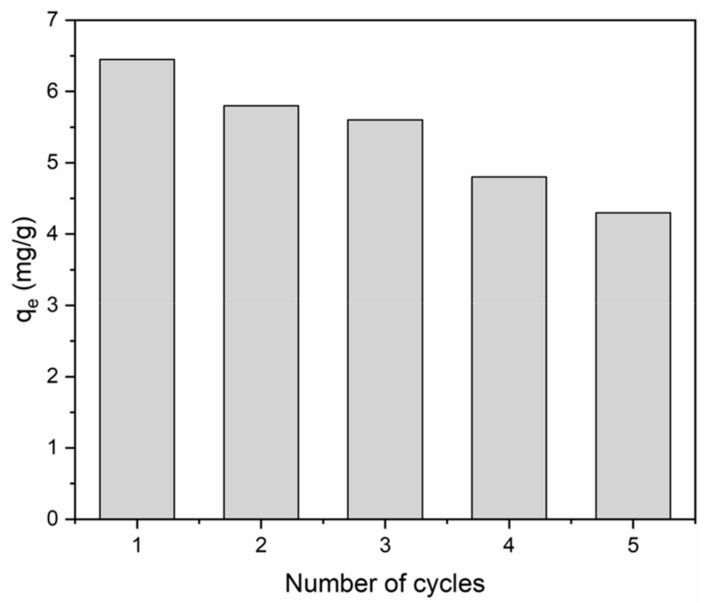
Reusability of BiFeO_3_/biochar coupled magnetic material for methylene blue removal (*V* = 0.05 L, *T* = 25 °C, *t* = 240 min, pH=6, *m* = 0.1299 g).

The regeneration and reuse capability of the BiFeO_3_/biochar coupled magnetic material was tested, and the results are shown in Figure 15. Over the course of five adsorption–desorption cycles of the BiFeO_3_/biochar coupled magnetic material for methylene blue removal, its adsorption capability decreased gradually. After the fifth cycle, BiFeO_3_/biochar coupled magnetic material maintained 66.7% of its original adsorption capability, which suggested its high reusability for methylene blue removal.

## 4. Conclusions

Chitosan effectively combines the properties of biochar and BiFeO_3_ materials and yields a strongly adsorbing magnetic composite that is easy to collect and recycle after adsorption. The amount of methylene blue adsorbed onto the BiFeO_3_/biochar coupled magnetic material increases with increasing initial concentration of methylene blue, reaction time, and temperature. The adsorption process, which involves both chemical and physical adsorption, improves under neutral and alkaline conditions and fits the pseudo-first-order kinetic model and Langmuir adsorption isotherm model. The BiFeO_3_/biochar coupled magnetic material provides a good adsorption capacity for methylene blue, reaching a maximum of 18.942 mg·g^−1^ at 25 °C.

## Figures and Tables

**Table 1:** Langmuir, Freundlich, and Temkin isothermal model parameters of methylene blue adsorption onto BiFeO_3_/biochar coupled magnetic material.

Isothermal Model	Parameter	Temperature (°C)		
		15	25	35
Langmuir	*q_max_* (mg·g^−1^)	10.727	18.942	14.287
	*K_L_* (L·mg^−1^)	0.036	0.018	0.070
	*R* ^2^	0.965	0.988	0.604
Freundlich	*n*	2.014	1.567	2.781
	*K_F_* (L·mg^−1^)	0.952	0.716	2.692
	*R* ^2^	0.936	0.988	0.581
Temkin	*a_T_* (L·g^−1^)	0.319	0.250	0.665
	*b_T_* (kJ·mol^−1^)	0.969	0.707	0.811
	*R* ^2^	0.975	0.965	0.601

**Table 2:** Maximum adsorption capacities of various adsorbents for methylene blue.

Adsorbent	Temperature (°C)	*q*_max_ (mg·g^−1^)	Reference
Chitosan-crosslinked bismuth ferrite/biochar	25	18.942	This study
Poorly crystalline hydroxyapatite	10	14.27	[50]
Biochar-supported hydroxyapatite	40	21.1	[51]
Raw kaolin	Room temperature	13.99	[52]
Magnetic multi-wall carbon nanotubes	25	15.87	[53]
Acrylic acid–acrylonitrile–N-isopropylacrylamide polymeric gels	25	2.79	[54]
Water-insoluble β-cyclodextrin polymer crosslinked by citric acid	30	13.8	[55]

**Table 3:** Thermodynamic parameters of methylene blue adsorption on BiFeO_3_/biochar coupled magnetic material.

Parameter	Temperature (°C)		
	15	25	35
ln *K*^0^	−1.118	−1.106	−0.381
Δ*G*^0^ (kJ·mol^−1^)	−0.783	−0.820	−1.748
Δ*H*^0^ (kJ·mol^−1^)	26.857		
Δ*S*^0^ (J·mol^−1^·K^−1^)	82.974		

**Table 4:** Pseudo-first-order and pseudo-second-order kinetic model parameters for methylene blue adsorption.

Adsorbent	Pseudo-First-Order			Pseudo-Second-Order		
BiFeO_3_/biochar coupled magnetic material	*q_e_*_,1_ (mg·g^−1^)	*k*_1_ (min^−1^)	*R* ^2^	*q_e_*_,2_ (mg·g^−1^)	*k*_2_ (g·mg^−1^·min^−1^)	*R* ^2^
	7.069	0.012	0.990	9.800	9.734 × 10^−4^	0.984
